# Addressing data imbalance in Sim2Real: ImbalSim2Real scheme and its application in finger joint stiffness self-sensing for soft robot-assisted rehabilitation

**DOI:** 10.3389/fbioe.2024.1334643

**Published:** 2024-06-14

**Authors:** Zhongchao Zhou, Yuxi Lu, Pablo Enrique Tortós, Ruian Qin, Shota Kokubu, Fuko Matsunaga, Qiaolian Xie, Wenwei Yu

**Affiliations:** ^1^ Department of Medical System Engineering, Chiba University, Chiba, Japan; ^2^ Institute of Rehabilitation Engineering and Technology, University of Shanghai for Science and Technology, Shanghai, China; ^3^ Center for Frontier Medical Engineering, Chiba University, Chiba, Japan

**Keywords:** imbalanced sim2real problem, scarce real-world data, CycleGAN, finger joint stiffness self-sensing technology, soft robot-assisted rehabilitation

## Abstract

The simulation-to-reality (sim2real) problem is a common issue when deploying simulation-trained models to real-world scenarios, especially given the extremely high imbalance between simulation and real-world data (scarce real-world data). Although the cycle-consistent generative adversarial network (CycleGAN) has demonstrated promise in addressing some sim2real issues, it encounters limitations in situations of data imbalance due to the lower capacity of the discriminator and the indeterminacy of learned sim2real mapping. To overcome such problems, we proposed the imbalanced Sim2Real scheme (ImbalSim2Real). Differing from CycleGAN, the ImbalSim2Real scheme segments the dataset into paired and unpaired data for two-fold training. The unpaired data incorporated discriminator-enhanced samples to further squash the solution space of the discriminator, for enhancing the discriminator’s ability. For paired data, a term targeted regression loss was integrated to ensure specific and quantitative mapping and further minimize the solution space of the generator. The ImbalSim2Real scheme was validated through numerical experiments, demonstrating its superiority over conventional sim2real methods. In addition, as an application of the proposed ImbalSim2Real scheme, we designed a finger joint stiffness self-sensing framework, where the validation loss for estimating real-world finger joint stiffness was reduced by roughly 41% compared to the supervised learning method that was trained with scarce real-world data and by 56% relative to the CycleGAN trained with the imbalanced dataset. Our proposed scheme and framework have potential applicability to bio-signal estimation when facing an imbalanced sim2real problem.

## 1 Introduction

In the field of deep learning, it is a prevalent method to train the model within a simulated environment and afterward deploy it in real-world scenarios (Tobin et al., 2017). However, modeling discrepancies between the simulation and real-world domains make it difficult to replicate simulation results in the real world (Truong et al., 2021). The gap between simulation and real-world data is known as the simulation-to-reality (sim2real) problem (Hofer et al., 2021). Additionally, in certain fields like medicine and healthcare, the challenge is not only addressing the sim2real problem but also doing it in the context of data imbalance, especially with featured abundant simulation data and scarce real-world data. This phenomenon arises because obtaining real-world data is expensive and risky, and in some cases, even illegal or unethical (Abascal et al., 2021). Such challenges can be further characterized as the imbalanced sim2real problem. Further refinement arises in the imbalanced sim2real problem depending on the nature of the real-world domain. When the real-world domain is a categorical variable, the problem is identified as a classification-type imbalanced sim2real problem. Conversely, when the real-world domain is continuous, it presents a specific challenge known as the regression-type imbalanced sim2real problem (Han et al., 2022). The regression-type imbalanced sim2real problem is particularly difficult because regression is equivalent to having theoretically infinite categories, demanding greater efficacy and accuracy in the transformation process. To bridge the gap between simulated and real-world environments with imbalanced data, researchers have proposed some methods, which are mainly categorized into domain randomization and domain adaptation (Salvato et al., 2021). However, both methods encounter challenges when confronted with the regression-type imbalanced sim2real problem.

Starting with domain randomization, it entails randomizing the simulation model to a wide range of simulated environments (such as the parameters of the friction and contact models and possible delays in the actuation) during training (Muratore et al., 2022). By training in such varied environments, models have the potential to attain superior generalization capabilities in the real-world (Tobin et al., 2017). Domain randomization has the potential to address the regression-type imbalanced sim2real problem completely, avoiding the dependence on the real-world data. However, domain randomization can lead to significant computational costs because of the need for multiple simulations to account for all environmental variations (Josifovski et al., 2022). Despite the deployment of a multitude of simulation environments, domain randomization still cannot fully capture the complexities and natures of the real world (Zhao et al., 2020). Furthermore, while real-world data may be scarce, they are not entirely absent. Thus, exclusive reliance on domain randomization may lead to underutilizing the real-world data (Ding et al., 2020).

Conversely, domain adaptation aims to align the disparity between domains such that the trained model in simulation can be generalized into the real-world domain, which entails the training of models utilizing a combination of simulation and real-world data (Peng et al., 2022). Some researchers combined domain adaptation with imbalanced learning. Such methods can tackle the label shift problem encountered during the training and testing phases, which can ensure that models maintain robust classification performance even when the distributions of the training and test datasets diverge (Zhu et al., 2022; Ding et al., 2023). Other imbalanced domain adaption methods emphasize addressing imbalances that frequently arise among different categories for classification tasks, regardless of data count or distribution (Kuang et al., 2022).

However, current existing imbalanced domain adaptation learning almost exclusively focuses on classification issues, with scarce solutions addressing the regression-type sim2real problem. Moreover, most imbalanced domain adaptation methods address the issue of quantity imbalances in input data. Given the overarching context of sim2real, simulations serve as inputs and real-world data act as the corresponding outputs (as labels), while finding a source and target domains with balanced label space is usually arduous or even impossible (Farahani et al., 2021). Lastly, in situations with quantitative imbalances, the unpaired simulation and real-world data emerge as a significant constraint in harnessing this methodology. Thus, current domain adaptation techniques remain insufficient at addressing the regression-type imbalanced sim2real problem comprehensively.

**FIGURE 1:**
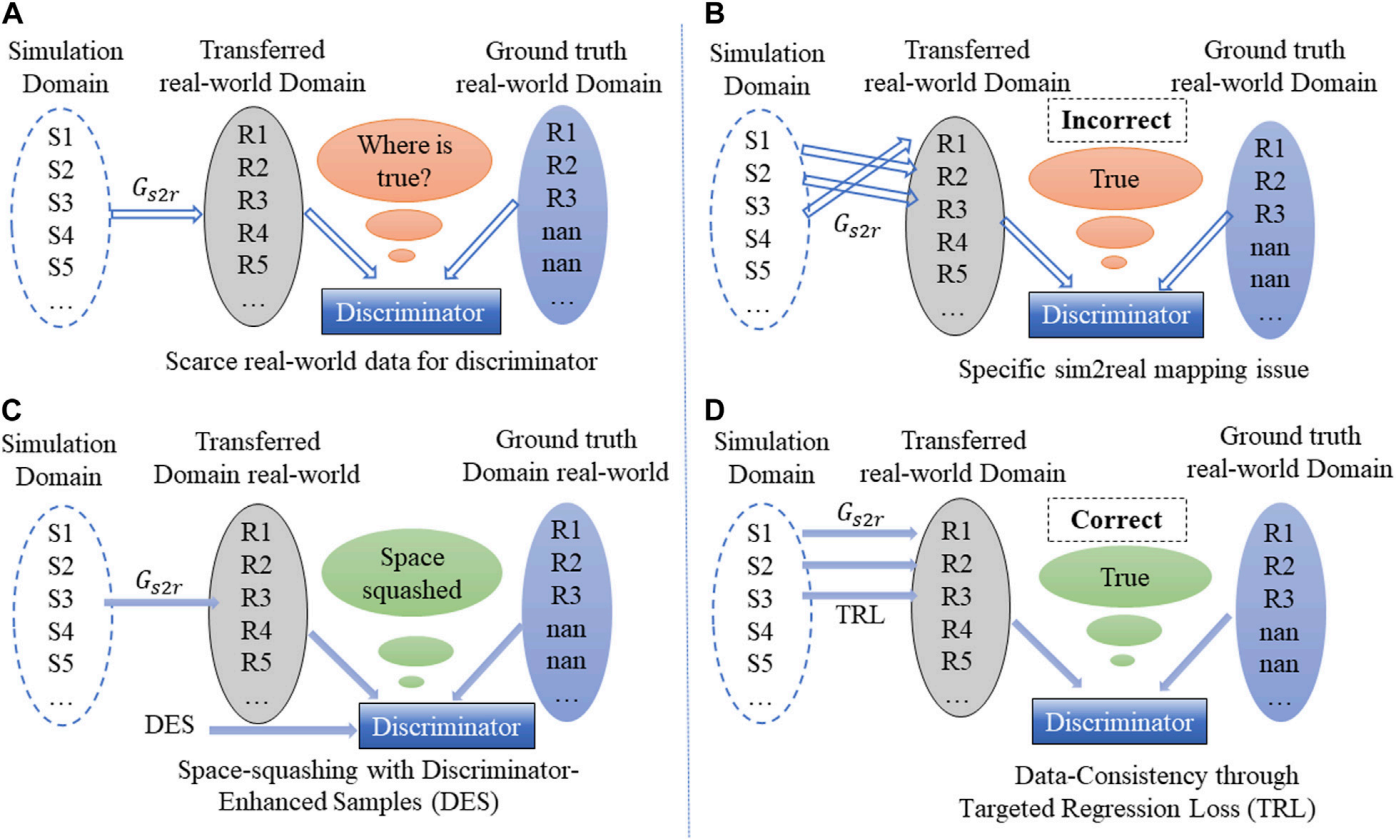
Current issues with CycleGAN when faced with the imbalanced sim2real problem and the potential solution. **(A)** Scarce real-world data for discriminator, **(B)** Specific sim2real mapping issue, **(C)** Space-squashing with DES, **(D)** Data-consistency through TRL.

Setting aside the imbalanced data factor, one methodology to address the sim2real challenge is the bidirectional unsupervised domain adaptation approaches, which entail the concurrent learning of both sim2real and real2sim mapping (Bhagat et al., 2019). Among these approaches, the cycle-consistent adversarial network (CycleGAN) stands out as one of the most notable approach (Zhu et al., 2017). The CycleGAN’s prowess lies in its ability to manage the sim2real challenge even with unpaired training datasets. Furthermore, it has been verified that simultaneously tackling both sim2real and real2sim not only enhances the quality of the generated data but also exhibits considerable generalizability even faced with unseen data samples. Consequently, this facilitates a more effective transfer of knowledge between the two domains (Chen et al., 2022). The efficacy has shown in scenarios where both simulation and real-world data are abundant and balanced, as demonstrated in works such as Chen et al. (2022), Jianu et al. (2022), and Zhao et al. (2023), which contain 1,429, 2079, and 1980 simulation and real-world data, maintaining a 1:1 ratio, respectively. However, when dealing with the regression-type imbalanced sim2real problem, CycleGAN may have the following two problems, as shown in Figures 1A, B:

1. Scarce real-world data for training discriminator. As shown in Figure 1A, in the CycleGAN framework for sim2real tasks, both the ground truth real-world domain and transferred real-world domain are typically provided to the discriminator. However, scarce real-world data make the training of discriminator quite difficult as it is difficult to obtain the correct distribution of real-world data, culminating in the generator’s incapacity to generate data that resemble real-world data.
2. Specific sim2real mapping issue. As shown in Figure 1B, while it is possible to learn a mapping from the simulation domain to the real-world domain with unpaired data using CycleGAN, it may not necessarily generate the desired specific mapping, causing the transferred real-world domain to deviate from the expectation (Harms et al., 2019). In other words, the structure of CycleGAN is capable of learning a domain transformation between simulation and real world represented by $G_{sim2real}$ (abbreviated as $G_{s2r})\;:x^{S}\to {\tilde{x}}^{S\to R}$ and $G_{real2sim}$ (abbreviated as $G_{r2s}$ ) $:x^{R}\to {\tilde{x}}^{R\to S}$ . Although we hope that $x^{S1}\to {\tilde{x}}^{S1\to R1}$ can be preserved, it potentially learns a domain transformation between *S1* and another real-world subdomain *R2* as well. Even though the discriminator may consider ${\tilde{x}}^{R1\;\text{or}\;R2\;or\;R3\;or\ldots }$ as “real,” such mappings do not align with our expectation (Zhang et al., 2018; Xie et al., 2020). This predicament stems from the CycleGAN’s inherent capability to ensure cycle consistency, e.g., $G_{r2s}$ ( $G_{s2r}\left(x^{S}\right)$ ) = $x^{S}$ . It is possible to demonstrate that any bijective geometric transformation T , along with its inverse ${T\;}^{-1}$ , can be applied to $G_{s2r}$ and $G_{r2s}$ such that ${G_{s2r}}^{\prime }$ = $G_{s2r}\;{}^{\circ}\;T$ and ${G_{r2s}}^{\prime }$ = $G_{r2s}\;{}^{\circ}\;{T\;}^{-1}$ and the transformed functions ${G_{r2s}}^{\prime }$ ( ${G_{s2r}}^{\prime }\left(x^{S}\right)$ ) = $x^{S}$ , is also cycle-consistent (here ° denotes the concatenation operation of two transformations). CycleGAN lacks the direct error between $G_{s2r}\left(x^{S}\right)$ and $x^{R}$ or $G_{r2s}\left(x^{R}\right)$ and $x^{S}$ , which introduce uncertainty and difficulty in achieving desired outputs task. Although the presence of abundant simulation and real-world data could somehow mitigate this issue, the results would be significantly affected when dealing with the quantitative regression-type sim2real problem with scarce real-world data.

To address the sim2real challenge with imbalanced paired–unpaired data, the imbalanced Sim2Real (ImbalSim2Real) scheme was proposed. Although the ImbalSim2Real scheme incorporates architectural components commonly found in models like the CycleGAN, such as $G_{s2r}$ and $G_{r2s}$ , as well as the discriminator for simulation data (abbreviated as $D_{sim}$ ) and discriminator for real-world data (abbreviated as $D_{real}$ ), it introduces several innovations tailored specifically to address the regression-type imbalanced sim2real problem.

**Separate training:** In the ImbalSim2Real scheme, in order to make full use of all real-world data, the dataset is segmented into paired and unpaired data, and two-fold training is performed.

Space squashing with discriminator-enhanced samples: The first-fold shifts attention to unpaired data. As shown in Figure 1C, to enhance the capability of the discriminator, the discriminator is space-squashed by providing additional discriminator-enhanced samples (DES). The strength of the discriminator plays a crucial role in the training of the generator as a stronger discriminator forces the generator to improve its ability to generate realistic samples (Schonfeld et al., 2020).

Data consistency through targeted regression loss: The second-fold training focuses on paired data. As shown in Figure 1D, to effectively utilize paired data and ensure a specific and quantitative mapping between the simulation domain and ground truth real-world domain, the second-fold training involves a data-consistency module, which is referred to as targeted regression loss (TRL). The update of $G_{s2r}$ is not only confined to the discriminator but also has a direct correlation with the TRL, hence lowering the uncertainty and difficulties associated with obtaining desired outputs. Following the enhancement by the first-fold, the second-fold was used to further minimize the solution space of $G_{s2r}$ for paired data in order to improve the accuracy of unpaired data generation.

As an application of the ImbalSim2Real scheme, we focus on the finger joint stiffness sensing problem, taken as a prime imbalanced sim2real problem in the soft robot-assisted rehabilitation scenario. Soft actuators have been applied in various healthcare fields, such as rehabilitation (Wang et al., 2021), surgery (Lu et al., 2023), and assistance (Zhou et al., 2022). In particular, Heung et al. analyzed the relationship between finger joint stiffness, soft actuator’s angle, and air pressure, and hence developed an accurate analytic angle-pressure-finger joint stiffness model (Heung et al., 2020). However, this approach is based on the chamber structure of the soft actuator, which cannot be used with a model-unknown soft actuator because of its model dependency (Matsunaga et al., 2023). In spite of the lack of the literature, it is not difficult to conceive the possibility of estimating finger joint stiffness by training a neural network-based finger joint stiffness self-sensing scheme with the air pressure and angle of a soft actuator as inputs. We utilized a simulation-based model, notably COMSOL Multiphysics for data collection. The simulation data would be transformed into real-world data through the ImbalSim2Real scheme, thus augmenting the availability of real-world data, and then, generated real-world data could be used to train a finger joint stiffness self-sensing scheme.

Our contribution can be summarized as follows:

1. An ImbalSim2Real scheme was specifically proposed for the purpose of transferring simulation data to real-world data in regression tasks with an imbalanced dataset.
2. A finger joint stiffness self-sensing scheme was proposed, in which the finger joint stiffness can be estimated online without the need for the analytic model of the soft actuator.
3. The novel finger joint stiffness self-sensing framework which combined the self-sensing scheme with the ImbalSim2Real scheme was proposed. To the best of our knowledge, this is the first framework for the learning-based estimation of finger joint stiffness. Furthermore, this framework holds potential for the application in other experiments requiring the estimation of biological signals in real world.

This paper is structured as follows. In Section 2, the proposed framework is presented and discussed. Section 3 provides a detailed description of the numerical imbalanced domain transfer experiment and finger joint stiffness experiment. The results of the experiments are presented and analyzed in Section 4. Ablation studies are conducted in Section 5 to further validate the efficacy of the proposed ImbalSim2Real scheme. In Section 6, the implications and limitations of the proposed framework are discussed. Finally, in Section 7, the conclusion and directions for future research are outlined.

## 2 Methods

### 2.1 Finger joint stiffness self-sensing framework

**FIGURE 2:**
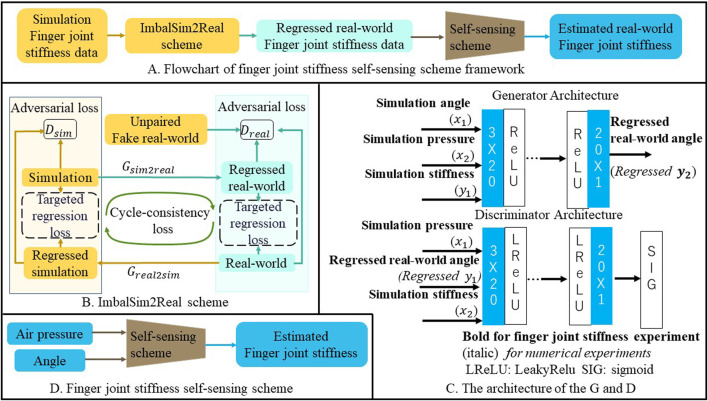
**(A)** A Flowchart of finger joint stiffness self-sensing scheme framework, **(B)** ImbalSim2Real scheme, **(C)** The architecture of the G and D, **(D)** Finger joint stiffness self-sensing scheme.

The flowchart of the finger joint stiffness self-sensing framework is depicted in Figure 2A, comprising both the ImbalSim2Real scheme and the self-sensing scheme. The input to the framework constitutes the simulated finger joint stiffness-related data (air pressure and angle). The simulated finger joint stiffness-related data were first transferred into regressed real-world finger joint stiffness data by the ImbalSim2Real scheme shown in Figure 2B. The regressed real-world finger joint data were then utilized to train the self-sensing scheme shown in Figure 2D, which could be applied to estimate real-world finger joint stiffness.

### 2.2 ImbalSim2Real scheme

As illustrated in Figure 2B, the ImbalSim2Real scheme comprises a generator $G_{s2r}$ that generated real-world data from simulation data and $G_{r2s}$ that generated simulation data based on real-world data (as mentioned before, the generated real-world data and generated simulation data were named regressed real-world data and regressed simulation data, respectively). A discriminator $D_{real}$ is applied for judging real-world data or regressed real-world data, and a discriminator $D_{sim}$ is applied for judging simulation data or regressed simulation data (all abbreviations for the symbols used are provided in Supplementary Material S1). The ImbalSim2Real scheme contained total three loss functions, namely, adversarial loss $L_{GAN}$ , targeted regression loss $L_{TRL}$ , and cycle-consistency loss $L_{cyc}$ . Given this diversity in loss mechanisms, the scheme employed a two-fold training approach.

#### 2.2.1 Adversarial loss

In this study, $L_{GAN}$ was divided into two parts, $L_{GAN\left(s2r\right)}$ and $L_{GAN\left(r2s\right)}$ , respectively. The adversarial loss is defined by Zhu et al. (2017), which is expressed as follows:

$$L_{GAN\left(s2r\right)}\left(G_{s2r},D_{real}\right)={\;E}_{real∼p_{data\left(real\right)}}\left[\log D_{real}\left(real\right)\right]+E_{sim∼p_{data}\left(sim\right)}\log\;((1-D_{real}\left(G_{s2r}\left(sim\right)\right),$$

$$L_{GAN\left(r2s\right)}\left(G_{r2m},D_{sim}\right)={\;E}_{sim∼p_{data\left(sim\right)}}\left[\log D_{sim}\left(sim\right)\right]+E_{real∼p_{data}\left(real\right)}\log\;\left((1-\right.D_{sim}\left(G_{r2s}\left(real\;\right)\right).$$

The real-world data conform to the distribution $p_{data}\left(real\right)$ , where $real∼p_{data\left(rea\right)}$ indicates that the real-world data are sampled from $p_{data}\left(real\right)$ . Similarly, the simulation data conform to the distribution $p_{data}\left(sim\right)$ , where $sim∼p_{data\left(sim\right)}$ indicates that the simulation data are sampled from $p_{data}\left(sim\right)$ . Although $L_{GAN}$ was the same as the loss function of CycleGAN, the training data were different. Whether in CycleGAN or GAN, the generator is trained to maximize the probability of the discriminator making a mistake, and the discriminator is trained to correctly classify the samples as real or generated, while the generator and discriminator perform a min–max game. At the same time, a powerful discriminator encourages the generator to increase its capacity to produce realistic samples, which is a critical factor in the training of the generator.

For $D_{sim}$ , because there is a large amount of true simulation data, $D_{sim}$ can make good judgments and $G_{r2s}$ can capture the simulation data distribution, regardless of the amount of real-world data. However, for $D_{real}$ , it can judge poorly when there are just a few true real-world data. In order to improve the performance of $D_{real}$ , a strategy of providing $D_{real}$ with DES was proposed. By providing $D_{real}$ with DES, the discriminator was exposed to a wider range of data. This squashed the solution space of the discriminator, which improved its ability to distinguish between real and fake samples, resulting in more accurate guidance to $G_{s2r}$ . Due to the inherent disparities between simulation environments and real-world data, a bijective relationship existed between simulation data and real-world data, while these two sets of data remained independent of each other. Consequently, this results in data derived from simulations being totally distinct from real-world data. Therefore, $G_{r2s}\left(G_{s2r}\left(\text{simulation}\right)\right)$ could be considered fake data for $D_{real}.$ After convergence, the data equivalency between simulation and $G_{r2s}\left(G_{s2r}\left(\text{simulation}\right)\right)$ would be established.

#### 2.2.2 Targeted regression loss

Only $L_{GAN}$ could hardly guarantee that the problem of mis-mapping would not arise for paired data. An example to illustrate the concept of the mis-mapping problem is shown herein. Consider two sets, X and Y, where each element in X has a corresponding element in Y based on a pre-defined mapping. Assume X = {1, 2, 3} and Y = {a, b, c}, with the mapping 1 → a, 2 → b, and 3 → c. When using the conventional GAN model to generate elements of Y from X, the generator may learn mappings such as 2 → a, 3 → b, and 1 → c, which do not only align with the specific desired mapping but also satisfies the GAN loss. In order to ensure data consistency, a specific loss term was introduced for paired simulation and real-world data, which can be expressed as

$$L_{TRL\left(s2r\right)}=\frac{1}{n}\sum_{i=1}^{n}{\left(G_{s2r}\left({sim}_{i}\right)-{real}_{i}\right)}^{2},$$

$$L_{TRL\left(r2s\right)}=\frac{1}{n}\sum_{i=1}^{n}{\left(G_{r2s}\left({real}_{i}\right)-{sim}_{i}\right)}^{2},$$

where i is the index of the data and *n* is the total amount of data. As depicted in Figure 2B, $L_{TRL}$ was employed to ensure the consistency of the regressed real-world and its corresponding real-world data. Similarly, $L_{TRL}$ could also be calculated when the real-world data were converted into regressed simulation data.

#### 2.2.3 Cycle-consistency loss

To solve the problem of training generator with unpaired data from two domains while preserving cycle consistency, CycleGAN introduced $L_{cyc}$ , which was defined as L1 loss in Zhu et al. (2017). In this study, we modified it to L2 loss, which was more suitable for regression tasks.

$$L_{cyc}\left(G_{s2r},G_{r2s}\right)={\;E}_{x_{sim}∼p_{data\left(x_{sim}\right)}}\left[{\left\|G_{r2s}\;\left(G_{s2r}\;\left(\text{sim}\right)\right)-\text{ sim}\right\|}_{2}\right]+{\;E}_{x_{real}∼p_{data\left(x_{real}\right)}}\left[{\left\|G_{s2r}\;\left(G_{r2s}\;\left(\text{real}\right)\right)-\text{ real}\right\|}_{2}\right].$$

#### 2.2.4 Two-fold approach training

Due to the presence of both unpaired and paired data types, the proposed ImbalSim2Real scheme required a two-fold approach for training the generator. The first-fold approach employed $L_{GAN}+L_{cyc}$ as the loss function. This approach used all the available data, including both paired and unpaired data. The second-fold approach utilized only the paired data and employed the use of $L_{TRL}$ as the loss function. The usage ratio of the first fold: second fold = 2:1.

### 2.3 Neural network implementation

As illustrated in Figure 2C, the generator utilizes a multilayer perceptron (MLP), consisting of 5 hidden layers with 20 nodes per layer, connected by an activation function ReLu and a linear layer. The activation function of the discriminator is LeakyReLu. It is worth mentioning that the output layer of the discriminator in the code is a linear layer rather than a sigmoid layer, which is used for calculating the least squares based on the framework of least squares generative adversarial net (LSGAN) to mitigate the issue of vanishing gradients (Mao et al., 2017). The Adam optimization algorithm is selected as the optimizer for all schemes. The finger joint stiffness self-sensing scheme also utilizes an MLP with five hidden layers, each containing 20 nodes. The activation function is ReLu, the output layer is a linear layer, and L2 loss is employed as the cost function. Throughout the training of all schemes, the Adam optimization algorithm is utilized. All schemes were developed using Python 3.9.12 64-bits. The neural networks were developed using PyTorch 1.12.1 with CUDA 11.3 support for enhanced computational performance.

## 3 Experiment setting

### 3.1 Numerical imbalanced domain transfer experiment setting

To investigate the capacity of the ImbalSim2Real scheme in addressing regression-type imbalanced sim2real problems with varying complexity domain transformations, three sets of numerical imbalanced domain transfer experiments (numerical experiments) were conducted between two domains: the source domain is represented by $y_{1}=f_{1}\left(x_{1},x_{2}\right)$ and the target domain is represented by $y_{2}=f_{2}\left(x_{1},x_{2}\right)$ . The functional relationships and respective data amount of the three sets are detailed in Table 1.

**TABLE 1:** Functional relationships for three groups.

Group	$y_{1}$	$y_{2}$	Range $x_{2}$	Number of $y_{1}$	Number of $y_{2}$	Paired data	Have $y_{1}$ no $y_{2}$	Have $y_{2}$ no $y_{1}$
A	$x_{1}x_{2}$	$x_{1}^{3}+2\sin\;\left(x_{2}\right)$	Random (10)	370	30	30	340	0
B	$x_{1}x_{2}$	$\sqrt{x_{2}}{-\sin\;(x}_{1})+\sqrt[{3}]{x_{2}}+{x_{1}}^{4}$	Random (20)	370	30	10	360	20
C	$x_{1}+x_{2}$	${{x_{1}x}_{2}\sin\;(x}_{1})+\left({x_{2}}^{3}-x_{2}\cos\;\left(x_{1}\right)\right)$	Random ( ± 10)	350	50	20	330	30

Group A involves a mapping from a complex domain to another complex domain, group B involves a mapping from a complex domain to a simple domain, and group C involves a mapping from a simple domain to a complex domain (the complexities of the three sets of numerical experiments are depicted in the Supplementary Material S2). The $x_{1}$ in each group ranges from −1 to 1, with a step size of 0.1. For $x_{2}$ , groups A, B, and C consist of random numbers drawn from a uniform distribution between (0, 10), (0, 20), and (−10,10) with four decimal places, respectively. All data were chosen randomly. Moreover, the number of data conformed to the following rules:

$$y_{1}\text{Number}=\text{Paired data}+\text{Have }y_{1}\text{ no }y_{2},$$

$$y_{2}\text{Number}=\text{Paired data}+\text{Have }y_{2}\text{ no }y_{1}.$$

Each group underwent an initial training phase of 5,000 epochs at a learning rate of 1e-4, followed by 5,000 epochs with a learning rate of 1e-5. The trained ImbalSim2Real scheme was compared to a supervised learning method trained on only paired data.

### 3.2 Finger joint stiffness experiment setting

#### 3.2.1 Data collection of finger joint stiffness data

In order to verify the effectiveness of the finger joint stiffness self-sensing framework, finger joint stiffness data in FEM software and real-world were collected.

**FIGURE 3:**
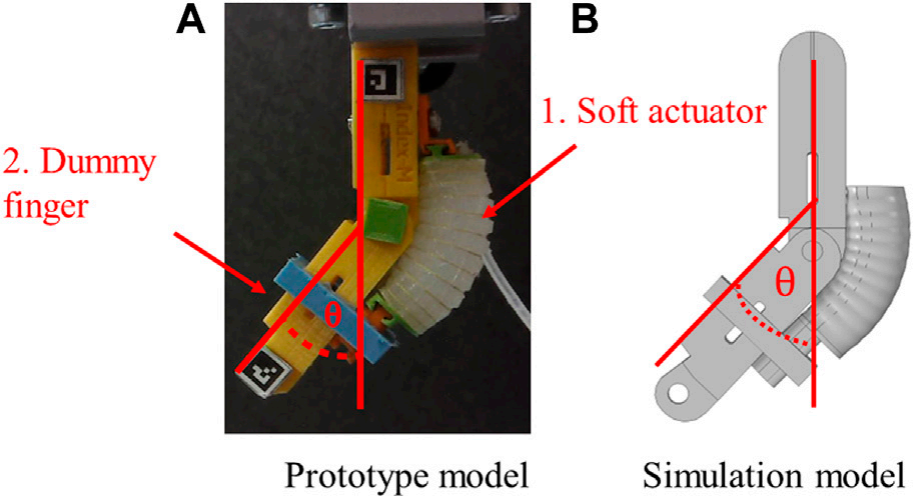
**(A)** Prototype model, **(B)** Simulation model.

The actuator’s body (shown in Figure 3(A-1)) was made entirely of Dragon Skin 10 MEDIUM (Smooth-On, Inc., US), with Kevlar™ (DuPont, Inc., US) wrapped around it using a two-dimensional hitching technique. The dummy joint (Figure 3(A-2)) was designed and created using a 3D printer with 20% density PLA based on our earlier work (Kokubu and Yu, 2020). The dimensions were based on the average size of Japanese index fingers (Department of Defense Human Factors Engineering Technical Advisory Group, 2000). Moreover, to represent finger joint stiffness, a torsion spring was integrated into the joint.

For the stationary finite element simulation, COMSOL Multiphysics^®^ was used, and all meshes were done using tetrahedral elements. Boundary load conditions were applied to simulate input air pressure and the hyperelastic behavior of the silicone sections, and a third-order Yeoh hyperelastic constitutive model was used, with the parameters obtained from the experimental data on Dragon skin in the Soft Robotics Materials Database Application (Marechal et al., 2021). The elastic band material, Smooth-on Sil 950, was modeled as a first-order Yeoh material model with C1 = 0.34 MPa (Labazanova et al., 2021) as it underwent less deformation than the actuator. To reduce computation time, symmetry boundary conditions were applied. Additionally, gravity was added in all models. The model was configured for measuring the bending angle (θ) (see Figure 3) based on experiments designed in the previous work (Tarvainen et al., 2018; Kokubu and Yu, 2020). The dummy joint was also modeled using solid mechanics interface and hinge joint conditions.

#### 3.2.2 Dataset preparation and training process setting

As shown in Figure 2C, the inputs of $G_{s2r}$ are simulation angle, pressure, and stiffness values, while the output is the regressed real-world angle. It should be noted that the simulation and real-world pressure are the same, with a maximum air pressure of 100 kPa and an increment of 5 kPa per pressure level. The finger joint stiffness data in the simulation were 0.11, 0.15, 0.2, 0.5, 0.58, 0.7, 1.03, 1.19, 1.4, 1.70, and 2.12 Nmm/° and in real world, were 0.11, 0.58, 1.03, 1.19, and 2.11 Nmm/°. These values were selected based on the range of finger joint stiffness observed in individuals with spasticity and in healthy individuals (Matsunaga et al., 2023). Among them, 0.11, 1.19, and 2.12 Nmm/° were selected as the training dataset, and 0.58, 1.53, and 1.03 Nmm/° were selected as the validation dataset. Notably, 1.53 Nmm/° was absent from both the simulation and real-world training datasets. Each dataset was divided into two sets, Dataset_1 containing only paired data, specifically the data on the stiffness values of 0.11, 1.19, and 2.12 Nmm/°, and Dataset_2 containing all the available data. Datasets were run alternately during the training process.

The ImbalSim2Real scheme was trained for a total of 17,000 epochs with a learning rate of 1e-4, followed by 17,000 epochs with a learning rate of 1e-5. The ImbalSim2Real scheme was compared to the supervised learning method training by Dataset_1 and the original CycleGAN training by Dataset_2. Following the successful training of the ImbalSim2Real scheme, all simulation data were input into the trained scheme to obtain regressed real-world data, which were subsequently utilized to train the finger joint stiffness self-sensing scheme for a total of 10,000 epochs, with a learning rate of 1e-4.

For comparative purposes, two additional finger joint stiffness self-sensing schemes were developed and implemented. The first alternative scheme entailed replacing the trained ImbalSim2Real scheme with the trained original CycleGAN, following the same steps as previously outlined. The second alternative scheme employed a supervised learning method, utilizing only real-world finger joint stiffness data.

## 4 Results

### 4.1 Numerical imbalanced domain transfer experiment results

The results of the numerical experiment are shown in Table 2, which indicate that the proposed scheme outperforms the supervised learning method in terms of validation MSE (detailed results are provided in Supplementary Material S3). Specifically, the proposed scheme decreases the validation MSE in groups A, B, and C by 3.81, 5.21, and 29.56 times, respectively.

**TABLE 2:** Total validation MSE of the basic numerical experiment.

Group	Supervised	Proposed	Supervised/proposed
A	1,092.6	286.66	3.81
B	313.9	60.2	5.21
C	8606479	291122	29.56

### 4.2 Finger joint stiffness experiment results

#### 4.2.1 Finger joint stiffness Imbalanced sim2real transfer results

**FIGURE 4:**
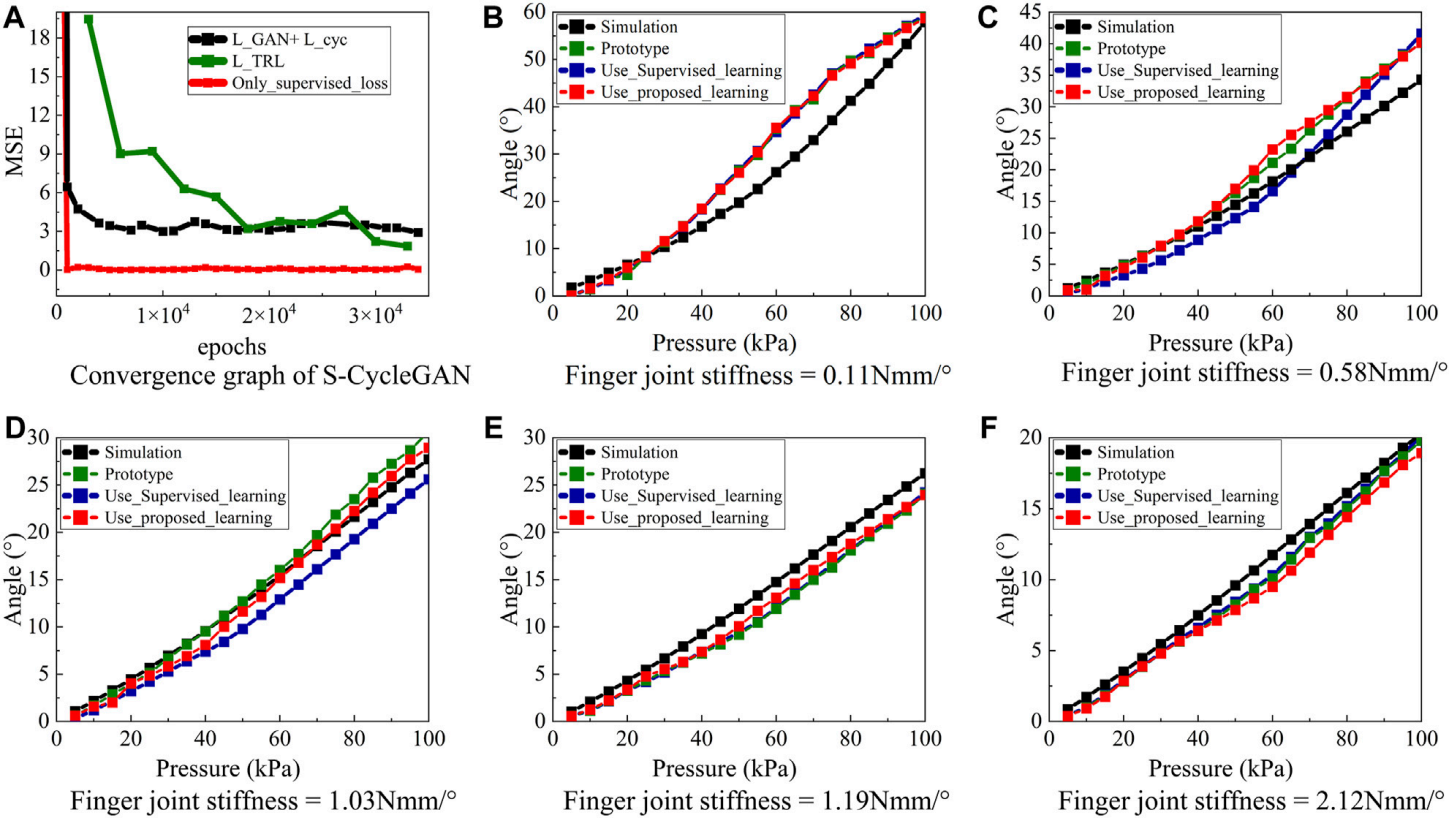
**(A)** Convergence graph, **(B)** Finger joint stiffness = 0.11 Nmm/°, **(C)** Finger joint stiffness = 0.58 Nmm/°, **(D)** Finger joint stiffness = 1.03 Nmm/°, **(E)** Finger joint stiffness = 1.19 Nmm/°.

The comparative analysis of training losses between the ImbalSim2Real scheme and the supervised learning method is illustrated in Figure 4A. The results demonstrate that both the supervised learning and the proposed ImbalSim2Real scheme achieve convergence, with the supervised learning method demonstrating a faster convergence speed. Moreover, as shown in Figures 4B–F, while the supervised learning method successfully transforms simulation data samples in the training dataset into real-world data, it is unable to perform such transformation when the simulation data samples are not in the training dataset. In contrast, the ImbalSim2Real scheme yields slightly poorer results for the data in the training dataset but better results for the data outside of the training dataset.

In addition, in cases where the finger joint stiffness values are 0.11, 0.58, and 1.03 Nmm/°, the angle values of simulation are smaller than the corresponding real-world values, but this is reversed for the stiffness values 1.19 and 2.12 Nmm/°. Since around 73% of the training dataset cases have simulation angles larger than their real-world counterparts, the supervised learning method tends to learn a pattern of reducing simulation angles for accurate real-world angle transformation. As a result, the resultant angle values for 0.58 and 1.03 Nmm/° are smaller than the corresponding angle values of simulation. On the other hand, the proposed ImbalSim2Real scheme is able to capture the true pattern of the real-world angles.

In order to quantitatively evaluate the efficacy of the ImbalSim2Real scheme, a success rate metric, defined by the proportion of regressed real-world angles falling within 10% of the true real-world angles, is used to gauge successful transformations. As shown in Table 3, the ImbalSim2Real method attains an overall success rate of 88%, which is considerably higher than 63% achieved by the supervised learning method and 41% by CycleGAN. Furthermore, in scenarios without applying any sim2real method, only 34% of the simulation data naturally align with the real-world data. Notably, for the data point of 1.03 Nmm/°, the simulation and real-world angles show close similarity in Figure 4D, and the supervised learning underestimates most angle values in simulation data in order to optimize the training loss. In contrast, the proposed scheme avoids this issue while maintaining data integrity. The results of the original CycleGAN demonstrate a relatively low success rate in transforming training data. In terms of generalizability, it performs somewhat better than supervised learning but is significantly less effective than the proposed ImbalSim2Real scheme.

**TABLE 3:** Successful rate for sim2real transfer for each method.

Stiffness value (Nmm/°)	0.11 (%)	0.58 (%)	1.03 (%)	1.19 (%)	2.12 (%)	Total (%)
Proposed	90	90	75	85	100	88
CycleGAN	40	30	10	80	45	41
Supervised	95	25	0	100	95	63
No transfer method	25	30	75	5	35	34

#### 4.2.2 Finger joint stiffness self-sensing framework results

The results presented in Table 4 support a similar conclusion: the finger joint stiffness self-sensing scheme trained through the Imbalanced sim2real scheme reduces the estimation error by 41% compared to the supervised learning method trained on only real-world data. Additionally, when replacing the Imbalanced sim2real scheme with CycleGAN, the estimation error increases by 56%. Moreover, the proposed framework exhibits smaller standard deviation (STD) values, indicating a more stable output. When compared to the average MSE of 0.15 Nmm/° reported in previous studies (Shi et al., 2020), supervised learning achieved a similar performance, and the proposed scheme is even better, providing further evidence of its effectiveness as a model-independent method.

**TABLE. 4:** Results for finger joint stiffness estimation for each method (mean ± STD).

Stiffness value (Nmm/°)	1.53	0.11	0.58	1.03	1.19	2.12	Total MSE	Average MSE
Supervised learning	1.12 ± 0.49	0.16 ± 0.21	0.38 ± 0.14	0.61 ± 0.20	1.23 ± 0.17	2.06 ± 0.20	16.00	0.13
CycleGAN	0.96 ± 0.25	0.22 ± 0.27	0.40 ± 0.08	0.59 ± 0.07	1.14 ± 0.11	1.67 ± 0.28	19.60	0.16
Proposed framework	1.32 ± 0.16	0.19 ± 0.19	0.59 ± 0.06	1.06 ± 0.12	1.47 ± 0.18	1.78 ± 0.33	9.43	0.07^a^

^a^ MSE of 0.15 Nmm/° reported in previous studies (Shi et al., 2020).

## 5 Ablation studies

To further assess the efficacy of the ImbalSim2Real scheme, ablation studies were conducted from three aspects: architectural differences, sensitivity to the ratio of paired *versus* unpaired data, and the impact of target domain data selection strategies.

### 5.1 Architectural analysis (via the group A dataset)

The ImbalSim2Real scheme was compared not only to the supervised learning method but also to the original CycleGAN and a variant of CycleGAN in which the discriminator only provided extra fake data without using a targeted regression loss (fake-provided CycleGAN).

**FIGURE 5:**
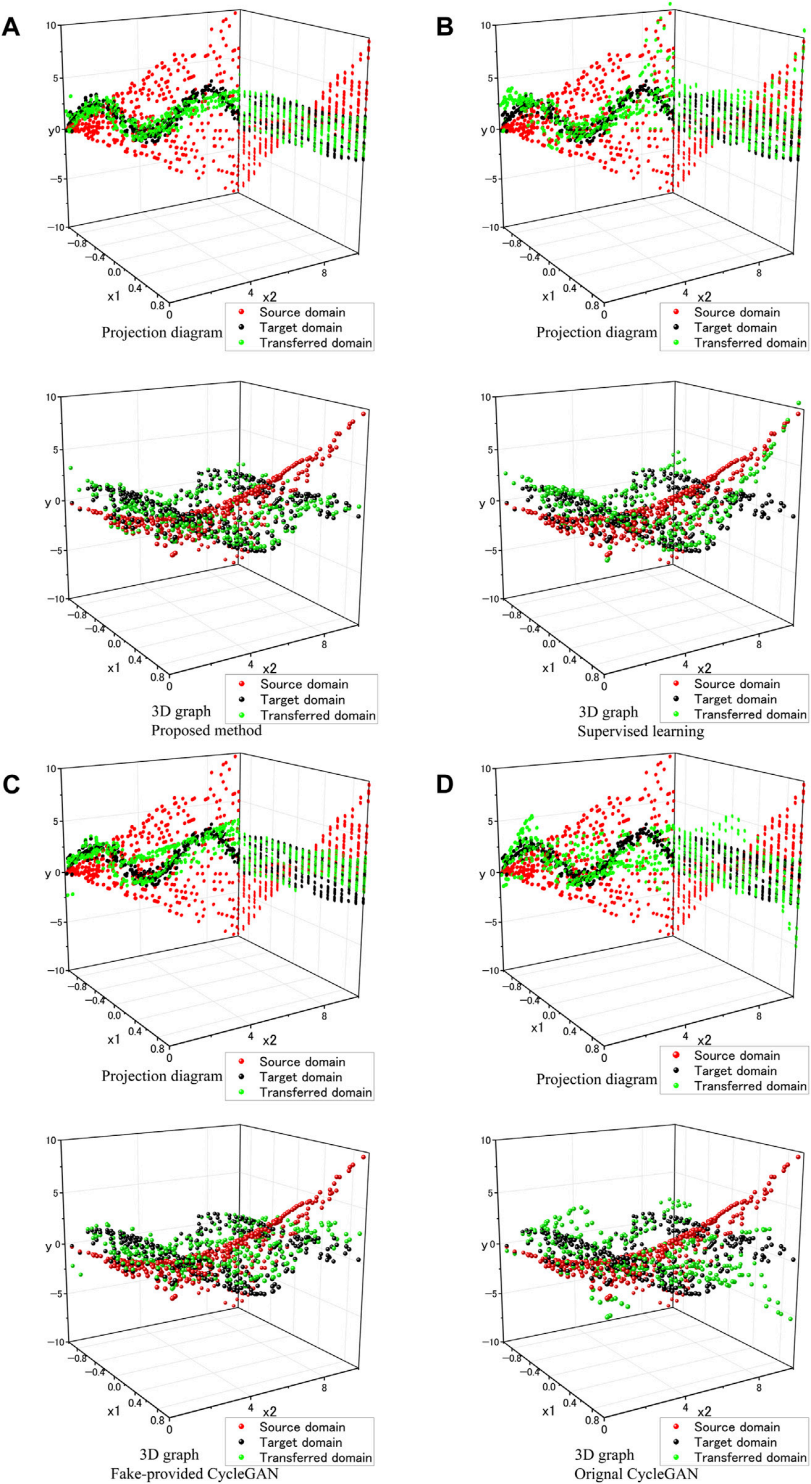
**(A)** Proposed method, **(B)** Supervised learning, **(C)** Fake-provided CycleGAN, **(D)** Original CycleGAN; each with upper: Projection diagram, lower: 3D graph.

The results in Table 5 reveal that the proposed ImbalSim2Real scheme has the smallest total validation MSE when converged. Meanwhile, Figure 5 presents 3D graphs of transformation (lower row) and projection diagrams (upper row) for different methods.

**TABLE 5:** Total validation MSE of group A for different methods.

	Proposed	Supervised	Fake-provided CycleGAN	Original CycleGAN	Original/fake-provided	Fake-provided/proposed	Original/proposed
MSE (unpaired)	284.8	1,092.2	468.3	1,244.8	2.65	1.64	4.37
MSE (paired)	1.9	0.381	29.7	28.6	0.96	15.63	15.05
Total	286.7	1,092.6	498.0	1,273.4	2.56	1.74	4.44

By comparing the $x_{2}-y$ plane of the projection diagrams in Figures 5B, D, despite both the supervised learning and the original CycleGAN exhibiting the same level of large validation MSE, the reasons are different. In Figure 5B, the transferred domain points in the $x_{2}-y$ plane are divergent when $x_{2}$ > 5. These points are considerably distant from the target domain points, thereby contributing to the large validation MSE. In contrast, the results of the original CycleGAN do not diverge. As shown in Figure 5D, in the $x_{2}-y$ plane, the transferred data points cluster around the target domain points but do not align with them when $x_{2}$ > 5. The large amount of such data results in a relatively large validation MSE.

The validation MSE in the fake-provided CycleGAN is smaller than that of the original CycleGAN in Table 5, upon comparing Figures 5C, D (the iterative process of the fake-provided CycleGAN is provided in Supplementary Material S4). It is evident that the fake-provided CycleGAN exhibits superior performance in terms of the domain transformation, which appears more compressed and adherent to the target domain points and compares to that of the original CycleGAN. This can be ascribed to the augmented number of fake data supplied to $D_{real}$ , which drives $G_{s2r}$ to generate data points that more resemble the target domain distribution. Nonetheless, an unambiguous line (green dots form a line from $x_{2}$ = 4–6.5) can be discerned in the projection diagram (Figure 5C), indicating suboptimal or incomplete training of $D_{real}$ in the corresponding region. Comparing the fake-provided CycleGAN method to the proposed scheme, it can be seen that the improvement in unpaired data is 1.54 times and that of paired data is 15.63 times.

### 5.2 Paired data sensitivity (via the group B dataset)

To investigate the significance of paired data for domain transformation and training convergence, we conducted experiments which maintained a constant total amount of $y_{2}$ data while controlling the ratio of paired data to unpaired $y_{2}$ data. Specifically, two ratios were considered, paired data: have $y_{2}$ no $y_{1}$ = 0:30 and 10:20. It should be noticed that the training process was performed 10 times for each ratio with 10,000 epochs.

The results in Table 6 indicate that when the data are entirely unpaired, the performance of the ImbalSim2Real scheme is significantly degraded. However, when the paired data points are introduced, the ImbalSim2Real scheme essentially converges to a similar state. Furthermore, we established a specific convergence success index, characterized as the validation MSE below 150, indicating convergence at the specific mapping. In the 0:30 case, due to the inability to use $L_{TRL}$ , only one group successfully converges at the specific mapping. For the remaining nine groups, although the training loss remains small, the validation loss is relatively large (additional results for different paired and unpaired ratios are provided in Supplementary Material S5).

**TABLE 6:** Validation MSE of group B for different methods (paired: unpaired).

	0:30	20:10
10 times average	353,220.35	81.67
10 times variance	4.87e + 11	1,384.25
Specific convergence success index	10%	100%

### 5.3 Data selection analysis (via the group C dataset)

To verify the impact of the dispersion of target domain data ( $y_{2}$ data) sampling on domain transformation, we performed experiments with extensive selection and intensive selection of $y_{2}$ data, respectively, while keeping the number of $y_{2}$ data and paired data constant (the specific details regarding the extent of the extensive and intensive selections are provided in Supplementary Material S6).

**FIGURE 6:**
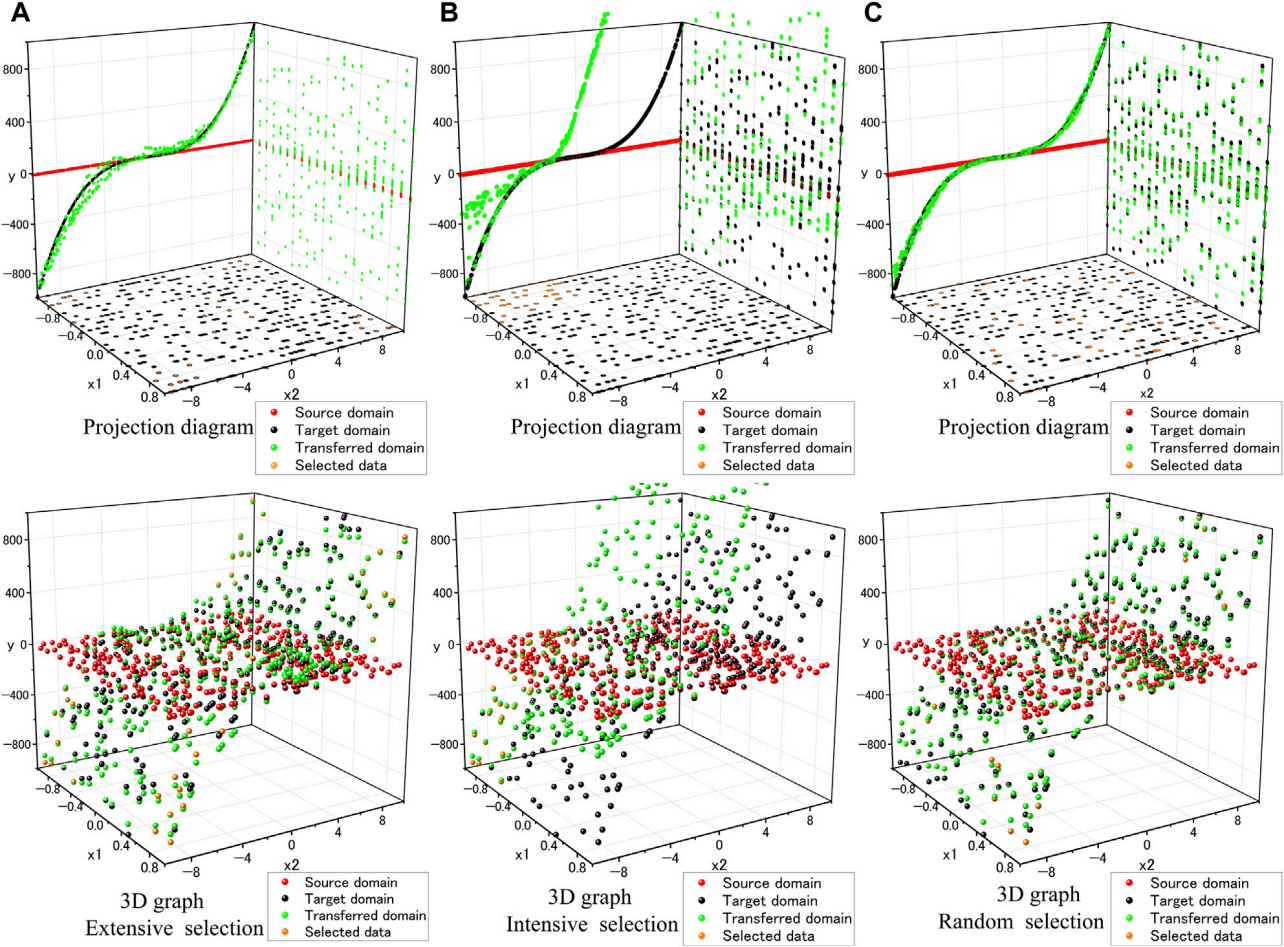
**(A)** Extensive selection, **(B)** Intensive selection **(C)** Random selection; each with upper: Projection diagram, lower: 3D graph.

The results in Figure 6B demonstrate that when the source domain is exceedingly simple and the sampled target domain data can only represent a fraction of the distribution, the ImbalSim2Real scheme may learn a distribution that passes through this partial representation. On the other hand, Figures 6A, C indicate that more extensive sampling can better reflect the overall distribution, which is advantageous for the training process of the ImbalSim2Real scheme. Group C inherently involves a transformation from a simple domain. The excessively simple distribution of the source domain (only a red line in Figure 6) results in a limited supply of fake data for $D_{real}$ , thereby limiting its capability to improve. In such cases, if the quality of the points collected by the source domain is poor, the learning effect of the uncollected points tends to be greatly reduced.

## 6 Discussion

The problem of regression-type imbalanced sim2real poses a significant challenge when deploying simulation-trained models to real world, particularly in fields with limited real-world data, such as medicine and healthcare. In this paper, we proposed the ImbalSim2Real scheme and conducted detailed comparisons with the supervised learning and the original CycleGAN through numerical imbalanced domain transfer experiments, finger joint stiffness experiments, as well as ablation studies.

### 6.1 Compared to the supervised learning

In both the numerical imbalanced domain-transfer experiment and the finger joint stiffness experiment, the ImbalSim2Real scheme was compared with the supervised learning method. The findings in Tables 2, 3, 5 consistently demonstrate that, regardless of the type of the domain transfer problem (complex-to-simple domain, complex-to-complex domain, and simple-to-complex domain), the proposed ImbalSim2Real scheme achieves a smaller total validation MSE than the supervised learning method. More specifically, the supervised learning exhibits a smaller validation MSE for paired data (within the training dataset) while showing a larger MSE for unpaired data (outside the training dataset). Figure 5B indicates that such low generalization is because the supervised learning methods only rely on paired data, thus struggling to accurately capture the target domain distribution, especially in cases of limited data quantity. The supervised learning method’s low data usage efficiency is the primary drawback when paired and unpaired data are included in the same training dataset. Additionally, as indicated in Figure 4A, the loss of the supervised learning method decreased rapidly, indicating a risk of overfitting, which also led to an increase in total validation MSE.

### 6.2 Compared to the original CycleGAN

In both the numerical imbalanced domain-transfer experiment and the finger joint stiffness experiment, comparisons were also made between the ImbalSim2Real scheme and the CycleGAN, with detailed contrasts explored in the ablation studies. According to Figure 5D, the original CycleGAN can learn a subset of the target domain distribution but still exhibits a large validation MSE. This phenomenon can be attributed to two key factors, which are identified as the ‘scarce real-world data for discriminator issue’ and the ‘specific sim2real mapping issue’.

To address the specific sim2real mapping issue, a specific mapping constraint $L_{TRL}$ was introduced to the ImbalSim2Real scheme. The results in Tables 5, 6 validate the effectiveness of this approach. In contrast to the supervised learning method, CycleGAN relies solely on the distribution information of paired data while entirely neglecting to utilize the critical aspect of accurate mappings between the source and target domains. This results in CycleGAN’s convergence being somewhat randomly achieved, which can be inferred through the convergence success index, as shown in Table 6. The ImbalSim2Real scheme combines the advantages of the supervised learning method and CycleGAN, effectively utilizing paired data through $L_{TRL}$ and $L_{GAN}$ while fully leveraging unpaired data via $L_{GAN}$ . Moreover, as shown in Figure 4A and Table 6, it is evident that not only is there no conflict between $L_{TRL}$ and $L_{GAN}$ + $L_{cyc}$ but this combination also contributes to more stable and robust convergence. $L_{TRL}$ represents a subset of the mapping sets that satisfies $L_{GAN}$ + $L_{cyc}$ ; thus, $L_{TRL}$ is expected to accelerate the convergence of $L_{GAN}$ + $L_{cyc}$ . Additionally, due to the alternating employment of $L_{GAN}$ + $L_{cyc}$ and $L_{TRL}$ in the ImbalSim2Real scheme, $L_{GAN}$ + $L_{cyc}$ serves as a regularization term to prevent overfitting caused by $L_{TRL}$ . Although the significance of $L_{TRL}$ and paired data was demonstrated in the paired data sensitivity experiment, the exact minimum proportion of paired data necessary for $L_{TRL}$ to exhibit its effectiveness remained unestablished, which needs to be investigated in future work.

To address the scarce real-world data for the discriminator issue, we proposed $D_{real}$ with additional DES. The effectiveness of this method is reflected in Table 5, where the validation MSE of the fake-provided CycleGAN is reduced by 2.65 times compared to the Original CycleGAN, even in the absence of $L_{TRL}$ . In instances, where there are paired data to guarantee that the data distribution has a specific limit, providing $D_{real}$ with additional DES enables it to determine which data distribution is fake, thereby further prohibiting $G_{s2r}$ from creating data comparable to the fake data distribution. This method cannot guarantee that the final $G_{s2r}$ distribution would be correct, but it increases the likelihood of learning a proper distribution compared to not providing DES. Furthermore, it is well-known that the training of GAN-type models is heavily dependent on the quality of the training data. For the ImbalSim2Real scheme, the results of the data selection analysis experiment (Figure 6) also demonstrate that intensive (this can be considered low-quality since the term “intensive” suggests a reduced likelihood of capturing the majority of distribution information) target domain sampling yields much worse results than extensive or random (high-quality) target domain sampling. Additionally, as DES are from the source domain, high-quality source domain data can also enhance $D_{real}$ 's performance. The appearance of an unambiguous line in Figure 5C substantiates this inference. A closer inspection of the source domain reveals the absence of sampling points in the region traversed by the unambiguous line within the $x_{2}-y$ plane. This deficiency in the distribution of source domain sampling points adversely affects $D_{real}$ 's training, reflecting in the observed anomaly. Similarly, the findings in Figure 6B also verify that when the source domain is inherently simple with limited information, providing additional DES did not yield effective results. Therefore, no matter whether there is an absence of certain source domain data or the source domain lacks complexity, both scenarios can lead to $D_{real}$ incapable of making accurate judgments. The final ImbalSim2Real scheme only misidentifies a domain that contains subsets similar to a portion of the target domain as the true target domain.

In summary, the resolution of the sim2real challenge hinges critically on both the quality and quantity of data from the source domain, as well as from the target domain. In the realm of the imbalanced sim2real problem, the limitations on the quantity of target domain data necessitate the use of higher-quality data, such as paired data or those capable of representing distribution characteristics. In cases where high-quality sampling of the target domain data is not feasible, it becomes essential to utilize the available target domain data with high quality, which is the primary motivation behind introducing $L_{TRL}$ . Regarding the source domain data, while increasing its quantity, high-quality source domain sampling points are critical for the successful training of $D_{real}$ . The ImbalSim2Real scheme effectively capitalizes on this characteristic, an aspect that is notably absent in the original CycleGAN architectures.

### 6.3 Application to the finger joint stiffness self-sensing framework

The proposed ImbalSim2Real scheme was used to resolve a practical issue in a model-independent finger joint stiffness self-sensing framework. The results of finger joint stiffness estimation in Table 4 demonstrate that the performance of the proposed framework is superior to the supervised learning method trained on a scarce real-world dataset and the original CycleGAN. However, the results of joint stiffness 1.19 and 2.12 Nmm/° are inferior to the supervised learning due to the following possible causes: first, during the training of the finger joint stiffness self-sensing scheme, real-world data are not used for training; instead, the training data consisted solely of regressed data, which included the values of 1.19 and 2.12mm/°. This implies that fine-tuning the trained model on real-world data may provide a potential solution to address this issue more effectively (real-world data fine-tuned results are provided in Supplementary Material S7). Second, 1.19 and 2.12 Nmm/° were the greatest values relative to the rest of the data; hence, their MSEs tend to be larger.

## 7 Conclusion

In this study, we proposed a novel ImbalSim2Real scheme. The proposed ImbalSim2Real scheme includes a targeted regression loss and augments fake data for enhanced domain transfer. This approach effectively leverages both paired and unpaired data to achieve a specific regression-type domain transfer, even in situations with limited available real-world data. Furthermore, we presented a finger joint stiffness framework as an application of the proposed ImbalSim2Real scheme. With the proposed framework, the validation loss for estimating real-world finger joint stiffness was reduced by roughly 41% compared to the supervised learning method and by 56% relative to the CycleGAN trained on the imbalanced dataset. Future research should concentrate on further enhancing the training effect of $D_{real}$ in the ImbalSim2Real scheme and investigating the potential applicability of the ImbalSim2Real scheme to high-dimensional datasets.

## Data Availability

The original contributions presented in the study are included in the article/Supplementary Material; further inquiries can be directed to the corresponding author.
