# Supplementary material for: Addressing data imbalance in Sim2Real: ImbalSim2Real scheme and its application in finger joint stiffness self-sensing for soft robot-assisted rehabilitation
Source: Front Bioeng Biotechnol. 2024 Jun 14;12:1334643. doi: 10.3389/fbioe.2024.1334643 (PMC11212110; doi:10.3389/fbioe.2024.1334643)
Supplement: Supplementary file 1 [file DataSheet1.docx]

Supplementary Material

# Supplementary Material 1 Summary of all data types

**Supplementary** Table 1. The description for each data

| Simulation data | The data from simulation |
| --- | --- |
| Real-world data | The data from real-world |
| Regressed real-world data | Simulation data 🡪 $G_{s2r}$ 🡪 **Regressed real-world data** |
| Regressed simulation data | Real-world data 🡪 $G_{r2s}$ 🡪 **Regressed simulation data** |
| Recovered simulation data | Simulation data 🡪 $G_{s2r}$ 🡪 Regressed real-world data  🡪 $G_{r2s}$ 🡪 **Recovered simulation data** |
| Recovered real-world data | Real-world data 🡪 $G_{r2s}$ 🡪 Regressed simulation data  🡪 $G_{s2r}$ 🡪 **Recovered real-world data** |
| True simulation data | The true data for $D_{sim}$ , which is the simulation data |
| Fake simulation data | The fake data for $D_{sim}$, which is regressed simulation data |
| True real-world data | The true data for $D_{\mathrm{real}}$, which means real-world data |
| Fake real-world data | The fake data for $D_{\mathrm{real}}$, which means regressed real-world data |

# Supplementary Material 2 The complexity of the three sets of numerical experiments

| Group | $y_{1}$ | $y_{2}$ |
| --- | --- | --- |
| A | $x_{1}x_{2}$ | $x_{1}^{3}+2\sin(x_{2})$ |
| B | $x_{1}x_{2}$ | $\sqrt{x_{2}}{-\sin(x}_{1})+\sqrt[3]{x_{2}}+{x_{1}}^{4}$ |
| C | $x_{1}+x_{2}$ | ${{x_{1}x}_{2}\sin(x}_{1})+({x_{2}}^{3}-x_{2}\cos(x_{1}))$ |


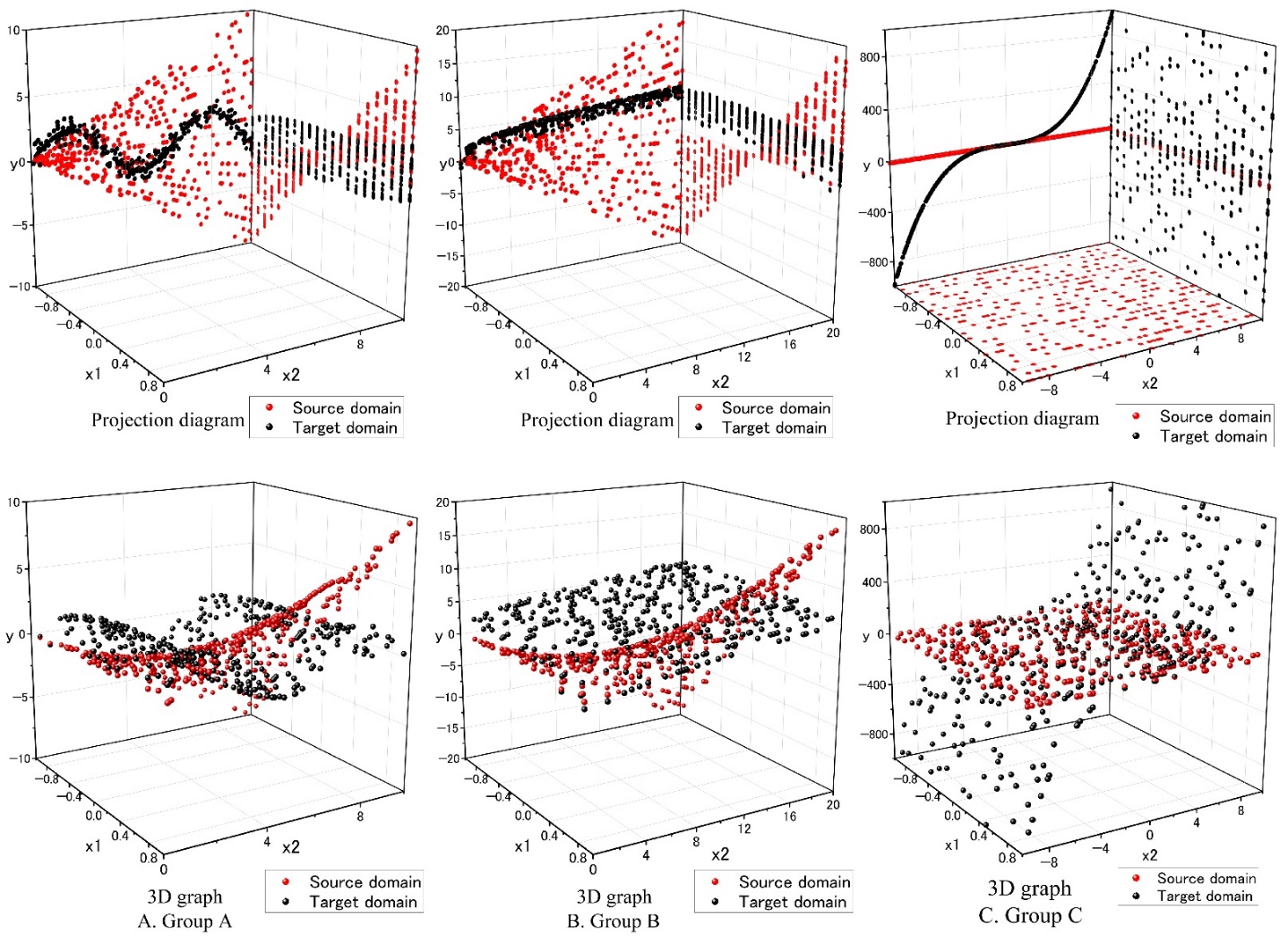


**Supplementary** Figure 1. Complexity of the three sets of numerical experiments

# Supplementary Material 3 detailed information and additional results for basic experiment


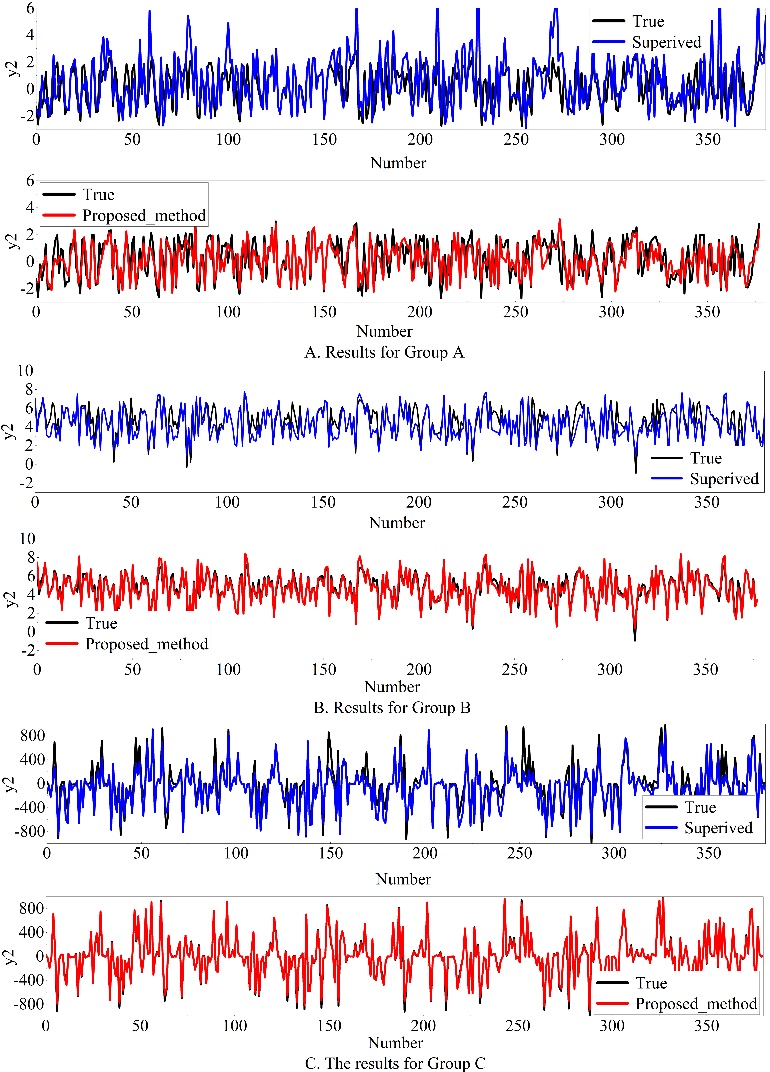


**Supplementary** Figure 2. The results of basic numerical experiment

# Supplementary Material 4 Iterative process of the Fake-provided CycleGAN


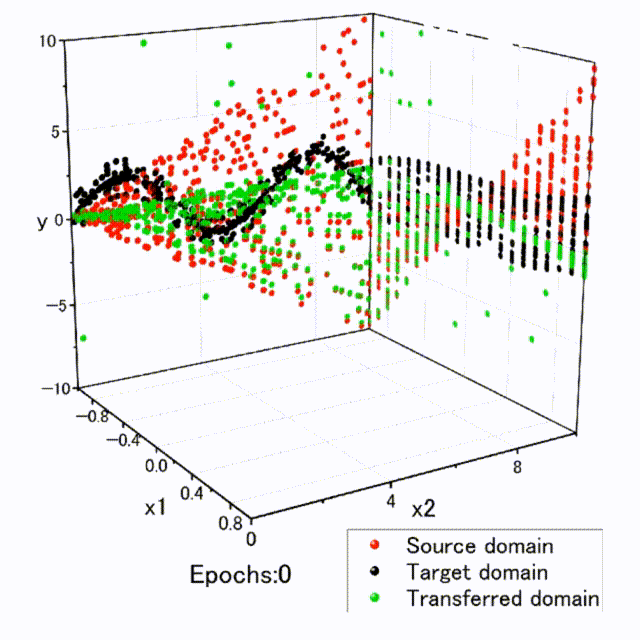
After 10,000 training iterations, we augmented the sampling points in the source domain by adding 40 points near the gaps. The iterative process of the Fake-provided CycleGAN was shown in **Supplementary** Figure 3. (A). We continued training the model for an additional 10,000 epochs based on the trained Fake-provided CycleGAN. The results demonstrated that the additional source domain sampling points compressed the previously existing line that crossed the blank areas. The final results were shown in **Supplementary** Figure. 3(B). This highlighted the importance of increasing the density of the source domain sampling points, as it could effectively improve the model's performance in sim2real tasks.

**Supplementary** Figure 3(A). Results of fake-provided after adding 40 points near the gaps

**Supplementary** Figure 3(B). Results of fake-provided after adding 40 points near the gaps


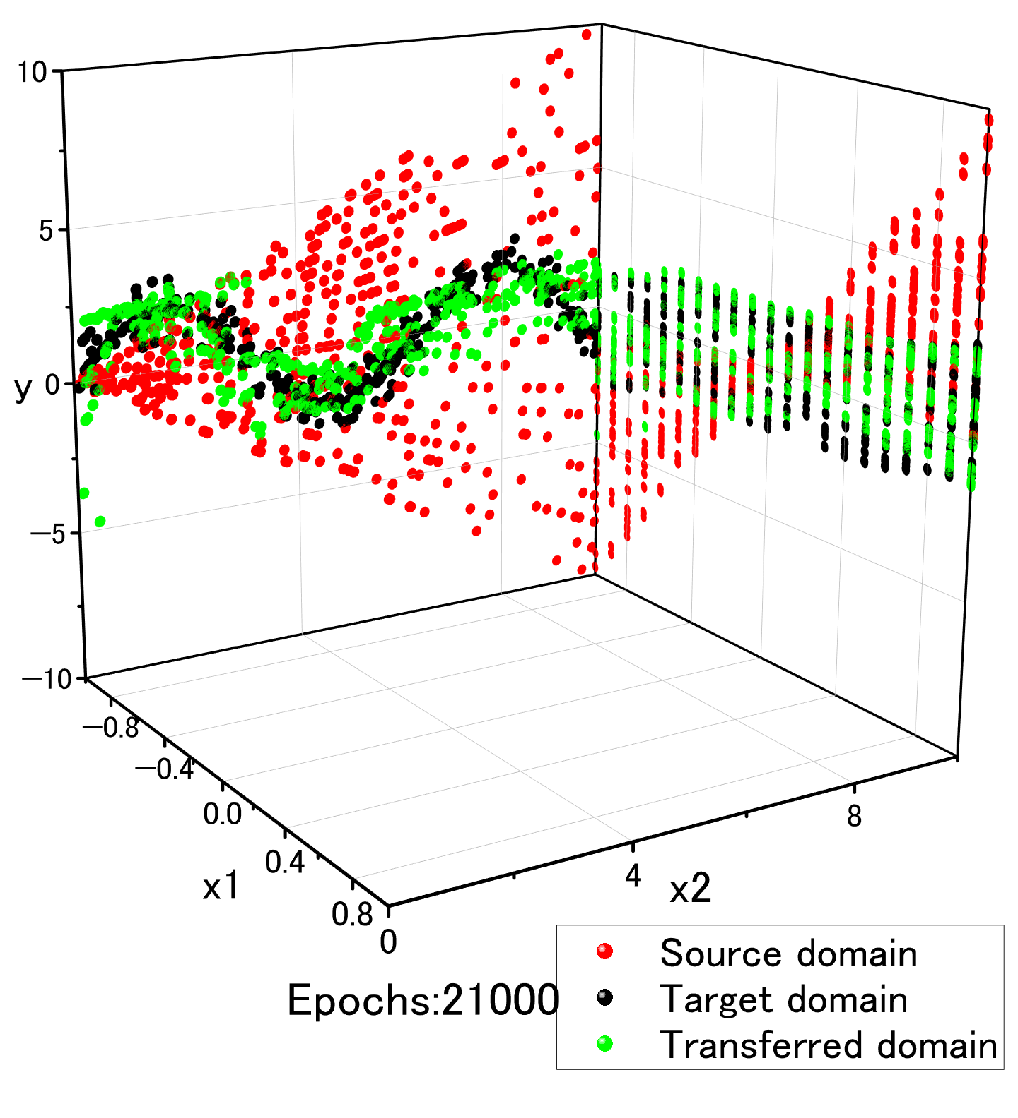


# Supplementary Material 5 Additional results for different paired-unpaired ratios

**Supplementary** Table 2. Validation MSE error for each group for additional experiment in Group B

|  | 0:30 (paired : unpaired) | 20:10 (paired : unpaired) |
| --- | --- | --- |
| Time 1 | 23412.43 | 99.20 |
| Time 2 | 7712.64 | 119.83 |
| Time 3 | 167.41 | 85.74 |
| Time 4 | 81.07 | 38.33 |
| Time 5 | 306.65 | 37.76 |
| Time 6 | 1378190.13 | 142.74 |
| Time 7 | 10503.87 | 89.07 |
| Time 8 | 16546.95 | 105.14 |
| Time 9 | 40025.38 | 34.29 |
| Time 10 | 2055257.00 | 64.60 |
| Average | 353220.35 | 81.67 |
| Variance | 4.87844e+11 | 1384.23 |

**Supplementary** Table 3. Validation MSE error for different paired-unpaired ratio for Group B

|  | 0:30 | 10:20 | 20:10 | 30:0 |
| --- | --- | --- | --- | --- |
| Total MSE for one time | 23412.43 | 60.17 | 99.20 | 40.63 |

# Supplementary Material 6 Extent of the extensive and intensive selections

**Supplementary** Figure 4. The extent of the extensive and intensive selections


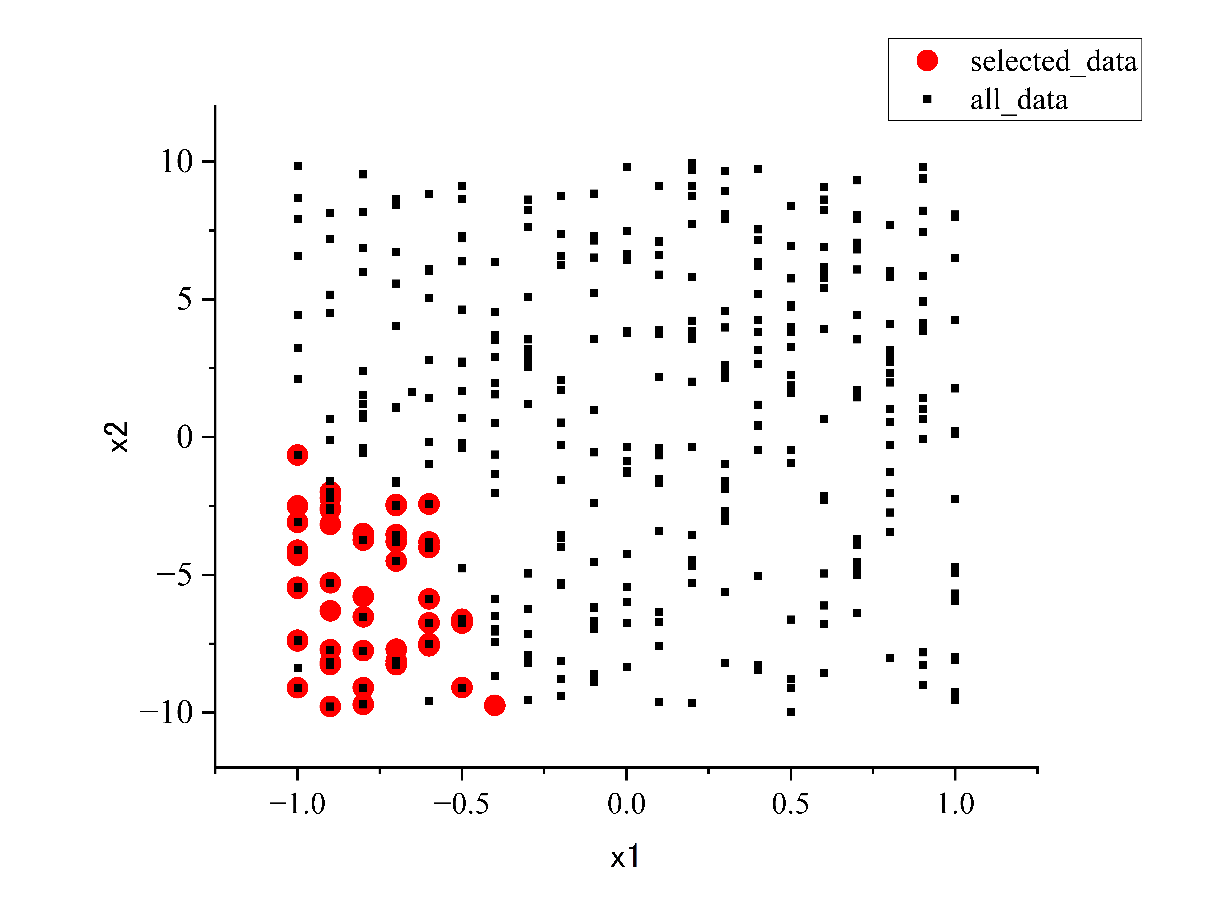


B. intensive selection


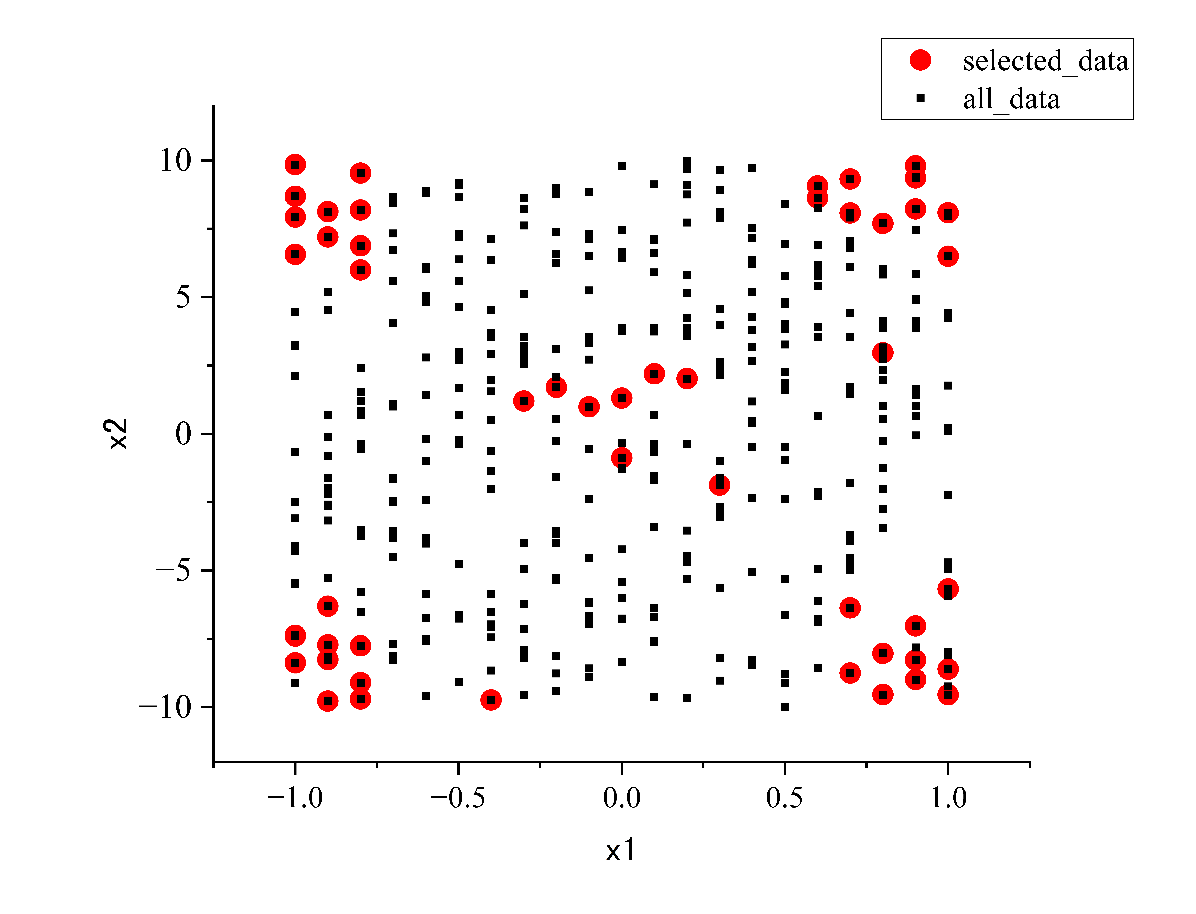


1. extensive selection

# Supplementary Material 7 Real-world data fine-tuned results

After training the finger joint stiffness self-sensing scheme using regressed real-world data, fine-tuning was performed using real-world data (0.11, 1.19, and 2.12 Nmm/°) on the finger joint stiffness self-sensing scheme. The results are shown in the **Supplementary** Table 4.

**Supplementary** Table 4. Results for finger joint stiffness estimation after fine-tuning(Mean$\pm$STD)

| Stiffness value (Nmm/°) | 1.53 | 0.11 | 0.58 | 1.03 | 1.19 | 2.12 | Total  MSE | Ave  MSE |
| --- | --- | --- | --- | --- | --- | --- | --- | --- |
| Supervised learning | 1.12  $\pm$0.49 | 0.16  $\pm$0.21 | 0.38  $\pm$0.14 | 0.61  $\pm$0.20 | 1.23  $\pm$0.17 | 2.06  $\pm$0.20 | 16.00 | 0.133 |
| Proposed framework | 1.32  $\pm$0.16 | 0.19  $\pm$0.19 | 0.59  $\pm$0.06 | 1.06  $\pm$0.12 | 1.47  $\pm$0.18 | 1.78  $\pm$0.33 | 9.43 | 0.078 |
| Real-world data  Fine-tuning | 1.32  $\pm$0.25 | 0.17  $\pm$0.15 | 0.62  $\pm$0.09 | 0.92  $\pm$0.14 | 1.29  $\pm$0.09 | 2.06  $\pm$0.16 | 4.57 | 0.038 |

The results demonstrated that the fine-tuned model achieved a reduction of 71.4% in validation loss compared to the supervised learning method.
